# Evidence of population structuring following population genetic analyses of *Fasciola hepatica* from Argentina

**DOI:** 10.1016/j.ijpara.2020.11.007

**Published:** 2021-05

**Authors:** Nicola J. Beesley, Elizabeth Attree, Severo Vázquez-Prieto, Román Vilas, Esperanza Paniagua, Florencio M. Ubeira, Oscar Jensen, Cesar Pruzzo, José D. Álvarez, Jorge Bruno Malandrini, Hugo Solana, Jane E. Hodgkinson

**Affiliations:** a Veterinary Parasitology, Institute of Infection, Veterinary and Ecological Sciences, University of Liverpool, Liverpool, UK; b Universidad de Los Lagos, Osorno, Chile; c Vicerrectoría de Investigación y Postgrado, Universidad Católica del Maule, Talca, Chile; d Departamento de Zoología, Genética y Antropología Física, Facultad de Biología, Universidad de Santiago de Compostela, Santiago de Compostela, Spain; e Laboratorio de Parasitología, Departamento de Microbiología y Parasitología, Facultad de Farmacia, Universidad de Santiago de Compostela, Santiago de Compostela, Spain; f Instituto de Investigación en Análisis Químicos y Biológicos (IAQBUS), Universidad de Santiago de Compostela, Santiago de Compostela, Spain; g Centro de Investigación en Zoonosis, Sarmiento, Chubut, Argentina; h Facultad de Ciencias Veterinarias, Universidad Nacional de la Plata (UNLP), La Plata, Argentina; i Cátedra de Enfermedades Parasitarias, Facultad de Ciencias Veterinarias, Universidad Nacional del Nordeste (UNNE), Corrientes, Argentina; j Laboratorio de Anatomía y Fisiología Animal, Facultad de Ciencias de la Salud, Universidad Nacional de Catamarca, San Fernando del Valle de Catamarca, Argentina; k Laboratorio de Biología Celular y Molecular, Centro de Investigación Veterinaria de Tandil (CIVETAN), CONICET, Facultad de Ciencias Veterinarias, Universidad Nacional del Centro de la Provincia de Buenos Aires (UNCPBA), Tandil, Argentina

**Keywords:** *Fasciola hepatica*, Population genetics, Population structure, Clones, Argentina

## Abstract

*Fasciola hepatica,* the liver fluke, is a trematode parasite that causes disease of economic importance in livestock. As a zoonosis this parasite also poses a risk to human health in areas where it is endemic. Population genetic studies can reveal the mechanisms responsible for genetic structuring (non-panmixia) within parasite populations and provide valuable insights into population dynamics, which in turn enables theoretical predictions of evolutionary dynamics such as the evolution of drug resistance. Here we genotyped 320 *F. hepatica* collected from 14 definitive hosts from four provinces in Argentina. STRUCTURE analysis indicated three population clusters, and principal coordinate analysis confirmed this, showing population clustering across provinces. Similarly, pairwise F_ST_ values amongst all four provinces were significant, with standardised pairwise F_ST_ (F′_ST_) ranging from 0.0754 to 0.6327. Therefore, population genetic structure was evident across these four provinces in Argentina. However, there was no evidence of deviation from Hardy–Weinberg equilibrium, so it appears that within these sub-populations there is largely random mating. We identified 263 unique genotypes, which gave a clonal diversity of 82%. Parasites with identical genotypes, clones, accounted for 26.6% of the parasites studied and were found in 12 of the 14 hosts studied, suggesting some clonemate transmission.

## Highlights

• 320 Argentinian *Fasciola hepatica* were genotyped using a panel of microsatellites.
• Overall there was high genotypic richness: 263 distinct genotypes were identified.
• Population structuring of *F. hepatica* was evident across Argentina.
• Within these sub-populations there is largely random mating.
• Transmission of clonemates occurs: clonal parasites accounted for 26.6% of all parasites.

## Graphical abstract

**Figure:**
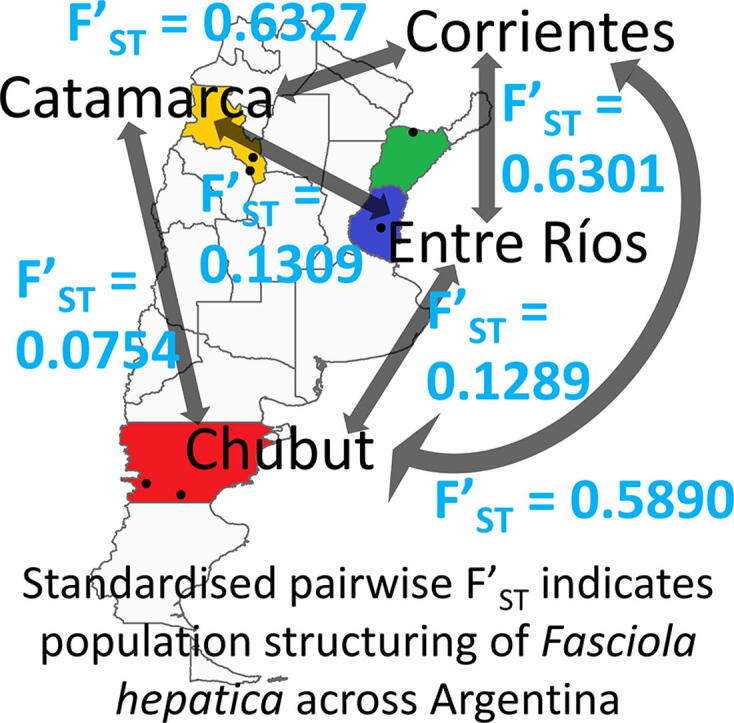

## 1 Introduction

The digenean trematode parasite, *Fasciola hepatica*, causes disease of economic importance in livestock worldwide (Mezo et al., 2011). An estimated 250 million sheep and 350 million cattle are at risk of infection (Hillyer and Apt, 1997). As a zoonosis, infection with *F. hepatica* is recognised as a neglected tropical disease by the World Health Organisation. Human infection is endemic in parts of South America, western Europe and Iran (Mas-Coma, 2005; World Health Organisation, 2007). In Argentina, prevalence of fasciolosis in cattle, based on the presence of eggs in faeces, has been reported to be as high as 77% (Moriena et al., 2004) and human endemic areas of fasciolosis have been detected in Argentina (Bargues et al., 2016). Drug resistance in *F. hepatica* is a substantial threat to the control of this parasite, in particular for the highly effective drug, triclabendazole (Kelley et al., 2016, Fairweather et al., 2020). Resistance was first reported in Australia more than two decades ago (Overend and Bowen, 1995) and is now widespread, including in Argentina (Olaechea et al., 2011), however we do not know the evolutionary dynamics of drug resistance development in *F. hepatica*. Population genetic studies can reveal the mechanisms responsible for genetic structuring (non-panmixia) within parasite populations and provide insights into population dynamics, which in turn enables theoretical predictions of evolutionary dynamics such as the evolution of drug resistance (Cornell et al., 2003, Schwab et al., 2006, Gorton et al., 2012).

Several studies exploring genetic structuring in populations of digenean trematodes have provided insights into life history characteristics such as modes of reproduction, life cycle patterns, use of multiple host species, and population sizes; and provide a useful basis to further understand the underlying mechanisms that may influence population structure in *F. hepatica*. In parasites with exclusively aquatic life cycles, it was proposed that the aquatic environment represents an ideal opportunity for dispersal and random mixing of parasites prior to infection of the definitive host, thus promoting panmixia and reducing co-transmission of clonemates generated by asexual reproduction in the intermediate host (a mollusc) (Criscione and Blouin, 2006). Empirical data has been generated that provides support for this theory, most often by studying the second intermediate host of different trematode parasites (e.g *Maritrema novaezealandensis*, Keeney et al., 2007a, Keeney et al., 2007b; *Coitocaecum parvum*, Lagrue et al., 2009; *Gymnophallus* sp., Leung et al., 2009; and *Proctoeces* cf. *lintoni*, Valdivia et al., 2014) and sometimes, the definitive host (e.g *Lecithochirium fusiforme*, Criscione et al., 2011). *Fasciola hepatica*, together with *Schistosoma mansoni,* are semi-terrestrial trematode/host systems, with one intermediate host (snail) and display reduced opportunity for dispersion of parasites as cercariae produced from asexual reproduction in the snail either infect the definitive host directly (*Schistosoma* spp.) or via vegetation (*F. hepatica*, as encysted metacercariae). There is evidence that such systems demonstrate low clonal diversities of 85% (305 of 360) and 80% (284 of 357) for *S. mansoni* parasites (Prugnolle et al., 2002, Prugnolle et al., 2004). A recent study in the trematode *Dicrocoelium dendriticum* (land snail-ant-ungulate) revealed the lowest clonal diversity for any trematode to date, with only 54 of 272 (19.9%) genotypes being unique. This observation was consistent with the restricted movements of the snails and ants under investigation, and the likely localised adherence of the slime balls, containing the cercariae, that infect the ant (Criscione et al., 2020). Appreciating the geographical scale over which studies are conducted facilitates their interpretation. For example, study of the snail intermediate host of the three-host trematode *Diplostomum pseudospathacerum* (snail-fish-bird), showed no genetic structure of parasites infecting snails over a large geographic range (>300 km), which the authors attributed to mitigation of any local effects of the snail, due to dispersion of the parasite by the highly motile bird definitive host (Louhi et al., 2010), whilst comparison of the three-host trematodes (two hosts of which are shared), *Coitocaecum parvum* and *Stegodexamene anguillae*, showed that population structures were dependent on the dispersal abilities of the most mobile host (a fish and an eel, respectively) (Blasco-Costa et al., 2012).

To appreciate the opportunities for disruption of panmixia and the role of random versus non-random transmission in *F. hepatica,* one has to consider the life cycle and dispersal of free-living stages (Gorton et al., 2012). Eggs are passed in the faeces of their definitive hosts; an individual parasite produces large numbers of eggs (up to 25,000 eggs per adult per day, Happich and Boray, 1969), which may or may not mix in the gallbladder prior to passing out in the faeces (Fairweather, 2011). The miracidium that hatches from the egg is motile and moves rapidly in localised water sources, but is relatively short lived and will die within a few hours if it does not penetrate the (semi-terrestrial) mud snail (typically *Galba* spp.) (Smith and Grenfell, 1984). Following the asexual development (effectively clonal expansion) that takes place within the snail (Thomas, 1883, Krull, 1941, Hodgkinson et al., 2018), the cercariae that are subsequently shed may have little opportunity for mixing as they rapidly encyst on vegetation, possibly aggregated in small clumps (Abrous et al., 2001). Therefore, it is possible that the transmission of parasites from asexual reproduction to the definitive host occurs over small scales, leading to the possibility of localised co-transmission of clonemates. Indications of clumped clonal transmission in sheep infrapopulations on a farm was reported by Vilas et al. (2012), with additional evidence for non-random transmission from the definitive to snail hosts, although higher clonal diversities ranging from 89-98% were also observed (Vilas et al., 2012, Beesley et al., 2017, Howell et al., 2020). There is some suggestion that infected *Galba truncatula* may disperse more and have higher dispersal survival than uninfected snails, however the authors considered it more likely that they were observing increased susceptibility of immigrant snails compared with local snail populations (Correa et al., 2017).

It is likely that, due to local and global movements of livestock and humans, the definitive host is the most mobile host, with the greatest opportunity for parasite mixing in *F. hepatica*, although this remains untested and is very much dependent on the extent of movement within countries and regions. For example, the median movement distance of cattle from one farm to another in Argentina is 140 km (range 9–2137 km), with most movements occurring within the same region (Aznar et al., 2011). Whilst existing studies provide some insight into genetic structuring in *F. hepatica* (Morozova et al., 2004, Semyenova et al., 2006, Walker et al., 2007, Walker et al., 2011, Teofanova et al., 2011, Vilas et al., 2012, Elliott et al., 2014, Thang et al., 2020), some studies lack power, or provide limited opportunities to infer life history characteristics. Given its complex life cycle, there is a need for extensive analysis of population genetic structure in *F. hepatica*. Here, we are interested in understanding population genetic structuring (disruption of panmixia) at a geographical level, determining the extent of clonal transmission, and identifying if there is evidence to support random (or non-random) mating at a local level within livestock, predominantly cattle, in Argentina.

## 2 Materials and methods

### 2.1 Populations of *F. hepatica*

**Fig. 1:**
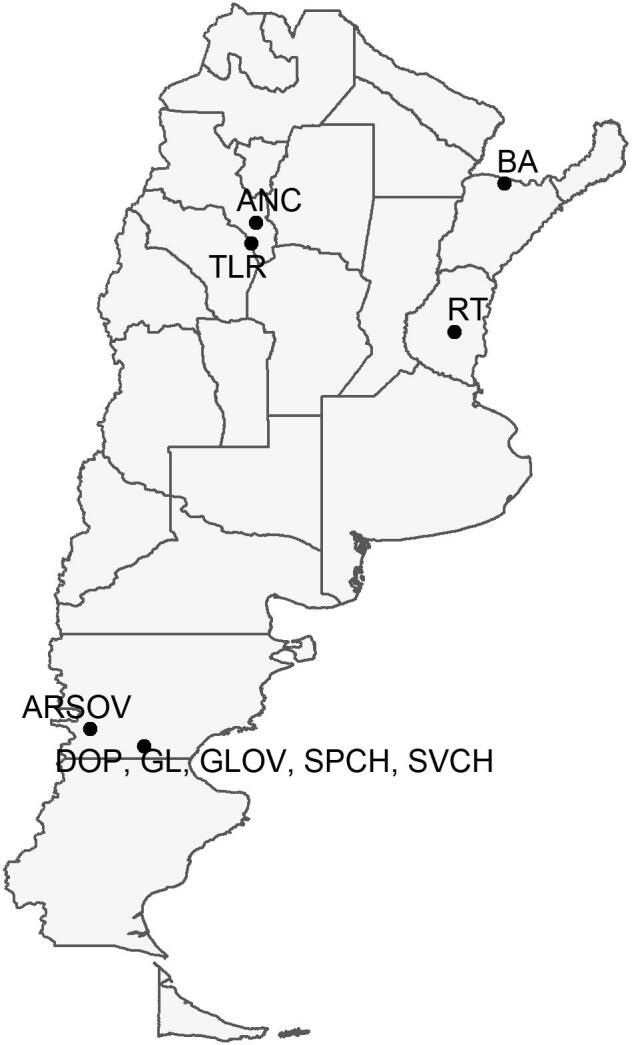
Provincial map of Argentina detailing the locations of sampling sites. Labels match those listed in Table 1.

A total of 338 adults of *F. hepatica* were collected from the livers of 14 naturally infected individual hosts, 11 cattle (*n* = 258), two sheep (*n* = 49) and one foal (*n* = 31), between April and September 2016 from four provinces in Argentina (Fig. 1). The animal identification, host species, number of parasites, and geographical origins are listed in Table 1; parasites collected from animals originating from the same farm have the same farm number. Parasites from each host were collected in plastic containers (Sigma-Aldrich, Spain), washed extensively in physiological saline (PanReac AppliChem, Spain) and fixed in 95% ethanol (Roche, Switzerland) until extraction of genomic DNA.

**Table 1:** *Fasciola hepatica* populations collected from sheep, cattle and a horse in Argentina.

Animal ID	Host	No. of parasites^a^	Farm No.	Locality	Province
ANC	Cow	14 (14)	1	Ancasti, Departamento Ancasti	Catamarca
TLR	Cow	13 (13)	2	Telaritos, Departamento Capayán	Catamarca
ARSOV	Sheep	24 (24)	3	Alto Río Senguer, Departamento Río Senguer	Chubut
DOP	Cow	14 (13)	4	Dorama Oporto, Departamento de Sarmiento	Chubut
GL1	Cow	12 (6)	5	Granja Lloyd, Departamento de Sarmiento	Chubut
GL2	Cow	20 (20)	5	Granja Lloyd, Departamento de Sarmiento	Chubut
GLOV	Sheep	25 (23)	5	Granja Lloyd, Departamento de Sarmiento	Chubut
SPCH	Cow	20 (18)	6	Colhué Huapi, Departamento de Sarmiento	Chubut
SVCH	Foal	31 (31)	7	Colhué Huapi, Departamento de Sarmiento	Chubut
BA1	Cow	28 (27)	8	Berón de Astrada	Corrientes
BA2	Cow	37 (35)	8	Berón de Astrada	Corrientes
RT1	Cow	12 (12)	9/10^b^	Rosario del Tala	Entre Ríos
RT2	Cow	48 (45)	9/10^b^	Rosario del Tala	Entre Ríos
RT3	Cow	40 (39)	9/10^b^	Rosario del Tala	Entre Ríos

^a^Number of parasites successfully genotyped at Fh_2, Fh_5, Fh_6, Fh_10, Fh_11, Fh_12, Fh_13 and Fh_15, and used in subsequent analyses, is shown in parentheses

^b^Parasites from Rosario del Tala were collected from two farms from the same locality; information on which animals originated from which farm was not available

### 2.2 Generation of a multilocus genotype (MLG)

In order to avoid contamination with eggs or sperm, a small section of each parasite anterior to the ventral sucker was used for DNA extraction. Samples were lysed in 300 μl of SSTNE extraction buffer (Blanquer, 1990. Phylogeographie intraspecifique d’un poisson marin, le flet *Platichthys flesus* L. (Heterosomata). Polymorphisme des marqueurs nucleaires et mitochondriaux. PhD thesis, University of Montpellier, France, for the preparation of 1L extraction buffer solution: 17.532 g of NaCl (PanReac AppliChem), 6.055 g of Tris (Sigma-Aldrich), 1 ml of EDTA 0.2 M (Sigma-Aldrich), 76.08 mg of EGTA (Sigma-Aldrich), 1 ml of spermidine (363 mg/5 ml H_2_0, −20 °C, Sigma-Aldrich), 1 ml of spermine (261 mg/5 mL H_2_0, −20 °C, Sigma-Aldrich), autoclave and store at −4°C) plus sodium dodecyl sulphate (0.1%, PanReac AppliChem) and 5 μl of proteinase K (20 mg/ml, Roche) for 3 h at 55 °C. After 20 min at 70 °C, samples were treated with 7.5 μl of RNase (10 mg/ml, Promega, Spain) for 1 h at 37 °C for RNA degradation. Total DNA was purified after protein precipitation (5 M NaCl) with freezing absolute ethanol (1 ml, Roche).

Each of the 338 *F. hepatica* samples (Table 1) was subjected to PCR and subsequent capillary electrophoresis to amplify a panel of 15 microsatellites (Cwiklinski et al., 2015). This panel has been used for population genetic analyses of *F. hepatica* from Great Britain (Beesley et al., 2017). PCR and capillary electrophoresis were carried out as described previously (Cwiklinski et al., 2015, Beesley et al., 2017), with the following modifications: (i) PCR products were diluted 50-fold prior to multiplexing 1 μl with 8.8 μl of formamide (Applied Biosystems, UK) and 0.2 μl of GeneScan 600 LIZ dye size standard v2.0 (Applied Biosystems) and (ii) the capillary sequencer used was an ABI 3500XL genetic analyser (Applied Biosystems) and fragment sizes were determined using 3500 Series Data Collection software 3.1 (Applied Biosystems).

### 2.3 Population genetic analyses

Allele calling from the electropherograms was successful for nine loci in the majority (92.9%) of parasites (Supplementary Table S1). Locus Fh_8 was excluded from analyses as we suspected the presence of null alleles. Any parasites with missing data at the other loci were also excluded. This left 320 parasites with an eight locus (Fh_2, Fh_5, Fh_6, Fh_10, Fh_11, Fh_12, Fh_13 and Fh_15) MLG for subsequent analyses (Table 1; Supplementary Table S1).

GenClone 2.0 (Arnaud-Haond and Belkhir, 2007, available from https://wwz.ifremer.fr/clonix/Logiciels/GenClone-2.0) was used to identify multicopy MLGs and calculate corresponding *P_sex_* values, adjusted using F_IS_ values (Parks and Werth, 1993). *P*_sex_ is the probability of observing *n* copies of an MLG in a sample size of *N* given sexual reproduction. If *P*_sex_ < 0.05 at *n* = 2 then all copies of that MLG can be considered to be a product of asexual reproduction (Gregorius, 2005). Before calculating *P*_sex_ values, preliminary analyses (STRUCTURE, principal coordinate analysis (PCoA) and pairwise F_ST_ – see below) were untaken to assess the population structure and decide at which sub-population level to assess *P*_sex_. For these preliminary analyses, all multicopy MLGs (≥2 parasites sharing an identical MLG based on eight loci) were initially assumed to be clonemates and to have arisen from asexual reproduction within the snail, and thus were reduced to one instance (261 parasites). The preliminary analyses indicated that *P*_sex_ should be assessed at the province level. Following analysis of *P*_sex_, only multicopy MLGs with a *P*_sex_ < 0.05 (i.e. those that were statistically significant) were reduced to one instance (263 parasites). Genotypic richness (Dorken and Eckert, 2001) was calculated for each province using the formula (G – 1)/(*N* – 1) [where G = the number of unique MLGs and *N* = the number of individuals]. The dataset of 263 parasites was used for the remaining analyses: STRUCTURE, PCoA, pairwise F_ST_, Hardy–Weinberg equilibrium, linkage disequilibrium, gene diversity.

STRUCTURE 2.3.4 (Pritchard et al., 2000, available from http://web.stanford.edu/group/pritchardlab/structure.html) was used to detect population structure. We trialled a number of different settings as suggested by Wang (2017). For the ancestry model, the admixture model was chosen which allows for individuals having mixed ancestry. As well as the default settings (uniform prior, same ALPHA for all populations, and an initial ALPHA value of 1) we also tested the alternative prior, allowed ALPHA to vary for each population, and reduced the initial ALPHA value to 1/K (0.0714). For the allele frequency model, we trialled both correlated and independent allele frequencies among populations (Wang, 2017). To determine the most appropriate value for K, STRUCTURE HARVESTER (Earl and vonHoldt, 2012, available from http://taylor0.biology.ucla.edu/structureHarvester/) was used to interrogate the results and calculate the mean and standard deviation Ln probability for each value of K. All the settings showed similar results with the mean of the estimated Ln probability peaking at the same value of K (Pritchard et al., 2000). We present the settings with the highest peak: admixture model (alternative prior, ALPHA allowed to vary for each population, and an initial value of ALPHA of 0.0714) with the correlated allele frequency model. Burn-in length was set at 500,000 and followed by 100,000 Markov Chain Monte Carlo repeats. K was set at 1 to 14 (the number of animals) and repeated 20 times. Results were plotted in R 3.6.1 (available from http://www.R-project.org/), using ggplot2 (Wickham, 2016).

We also assessed population structure using a multivariate method, PCoA, in Genalex v6.51b2 (Peakall and Smouse, 2006, Peakall and Smouse, 2012). A pairwise genetic distance matrix between each individual parasite was calculated and then PCoA was performed using the standardised covariance method. Pairwise F_ST_ values between parasites from the different provinces and corresponding *P* values were calculated using FSTAT 2.9.3.2 (Goudet, 1995, available from https://www2.unil.ch/popgen/softwares/fstat.htm) and Arlequin 3.5.1.3 (Excoffier and Lischer, 2010 available from http://cmpg.unibe.ch/software/arlequin35/). Multiple comparisons were addressed using the sequential Bonferroni method (Holm, 1979). Since high within-population genetic diversity will reduce the maximum value that F_ST_ can reach, F_ST_ was standardised (F′_ST_) using RecodeData (Meirmans, 2006, available from http://www.patrickmeirmans.com). The initial F_ST_ was divided by the maximum value of F_ST_ given the present within-population variance (Meirmans and Hedrick, 2011).

The number of different alleles and genotypes at each locus was calculated within each province and globally using GENEPOP 4.2.1 (Rousset, 2008, available from http://kimura.univ-montp2.fr/~rousset/Genepop.htm). Gene diversity was calculated using FSTAT 2.9.3.2 (Goudet, 1995); values were averaged across all loci for each province. To determine if there were deviations from Hardy–Weinberg equilibrium, F_IS_ and a corresponding one-tailed *P* value were calculated across all loci for each province using FSTAT 2.9.3.2 (Goudet, 1995). Unphased pairwise linkage disequilibrium tests for all pairs of loci in each province were performed using Arlequin 3.5.1.3 (Excoffier and Lischer, 2010). The number of permutations was set at 20,000 and the number of initial conditions for the Expectation Maximisation (EM) algorithm was set at five. There are 28 pairs of loci to test within each province; if four or more of these are significant (*P* < 0.05) this would be a sign that there is some level of linkage disequilibrium (cumulative binomial probability = 0.049; Waples, 2015).

### Ethical approval

Adult *F. hepatica* samples were recovered during post mortem examination of slaughtered animals by veterinary officers. No experiment was conducted on live animals. Ethical approval was not required under national laws of Argentina.

### 2.5 Data availability

Data supporting the conclusions of this article are included within the article. Genotypes from all parasites are available in Supplementary Table S1. Raw data is available from the corresponding author on request.

## 3 Results

### 3.1 Evidence of population structure of *F. hepatica* collected across Argentina

**Fig. 2:**
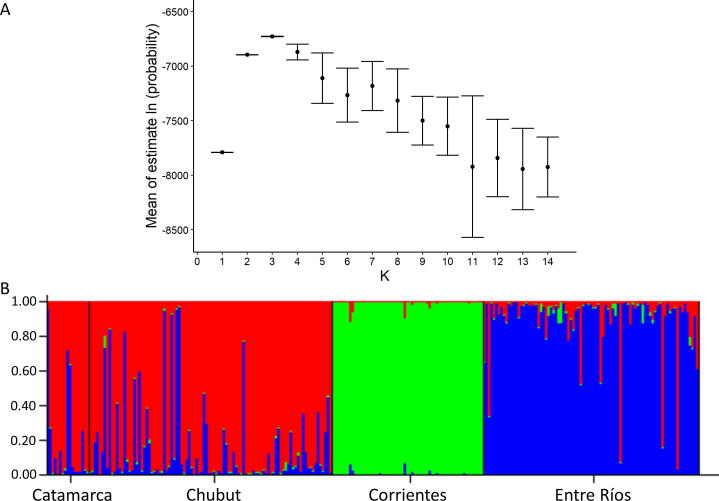
Results from Structure 2.3.4 (Pritchard et al., 2000), the admixture model (alternative prior, ALPHA allowed to vary for each population and an initial value of ALPHA of 0.0714) with the correlated allele frequency model was run 20 times for *K* = 1 to 14. Burn-in length was 500,000 followed by 100,000 Markov Chain Monte Carlo repeats. (A) The mean ± standard deviation of the estimated Ln probability is plotted for each value of *K*. The peak is at *K* = 3. (B) Bar plot from Structure 2.3.4 for *K* = 3. Results are grouped by province. There is strong clustering by region: parasites from Corrientes, parasites from Entre Ríos, and parasites from Catamarca and Chubut.

**Fig. 3:**
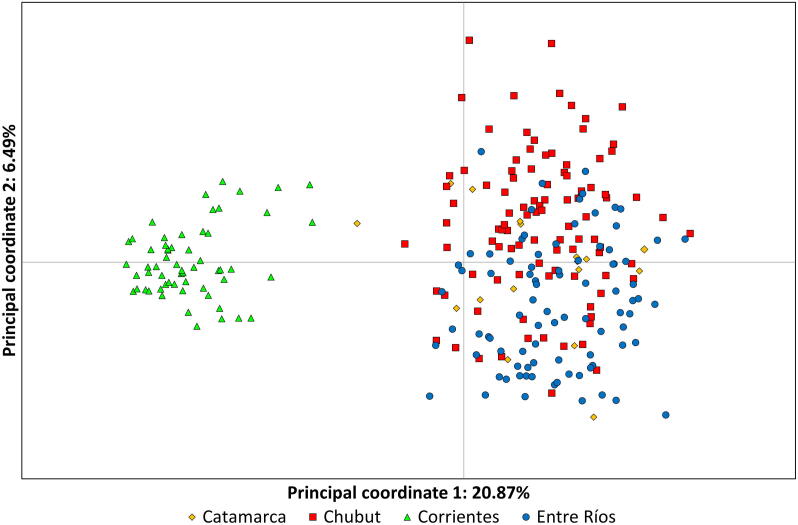
Result of principal coordinate analysis (PCoA) based on a pairwise genetic distance matrix between each individual parasite and the standardised covariance method was performed in Genalex v6.51b2 (Peakall and Smouse, 2006, Peakall and Smouse, 2012). PCoA analysis also shows population structure; this is particularly evident in parasites from Corrientes (triangles), however parasites from Chubut (squares) and Entre Ríos (circles) also show separation on axis 2.

STRUCTURE, PCoA and pairwise F_ST_ all support the conclusion that there is population structuring of *F. hepatica* across these four provinces of Argentina. STRUCTURE analysis indicates K = 3 as the most likely number of population clusters; this is the value of K at which the mean estimated Ln probability peaks (−6728.3; S.D. = 1.76) and plateaus (Pritchard et al., 2000; Fig. 2A). The iteration with the highest estimated Ln probability was chosen and a bar plot produced of the population clusters (Fig. 2B). There is strong clustering by region: parasites from Corrientes, parasites from Entre Ríos and parasites from Catamarca and Chubut. Similarly, PCoA analysis (Fig. 3) indicates population structure; this is particularly evident in parasites from Corrientes, however parasites from Chubut and Entre Ríos also show separation on axis 2 (Fig. 3). Further support for the STRUCTURE and PCoA analyses is seen based on pairwise F_ST_, with parasites from Corrientes showing the greatest differentiation. Furthermore, pairwise F_ST_ values amongst the four regions were significant (Table 2). When F_ST_ was standardised (F′_ST_; Table 3) genetic differentiation was apparent amongst all four regions, with the values largely consistent with results from STRUCTURE and PCoA.

**Table 2:** Pairwise F_ST_ results and corresponding *P* values between *Fasciola hepatica* parasites from each province studied in Argentina.

Province	Catamarca	Chubut	Corrientes	Entre Rios
Catamarca	0	0.0030	0.0000	0.0000
Chubut	0.0200 ^a^	0	0.0000	0.0000
Corrientes	0.3471 ^a^	0.2666 ^a^	0	0.0000
Entre Rios	0.0350 ^a^	0.0339 ^a^	0.2897 ^a^	0

F_ST_ values are below the diagonal and *P* values are above the diagonal.

^a^Significant differences based on the sequential Bonferroni method (Holm, 1979).

**Table 3:** Standardised pairwise F_ST_ (F′_ST_) results between *Fasciola hepatica* parasites from each province studied in Argentina.

Province	Catamarca	Chubut	Corrientes	Entre Rios
Catamarca	0			
Chubut	0.0754	0		
Corrientes	0.6327	0.5890	0	
Entre Rios	0.1309	0.1289	0.6301	0

F′_ST_ were calculated by dividing the initial F_ST_ by the maximum value of F_ST_ given the present within-population variance (Meirmans and Hedrick, 2011).

### 3.2 Genetically identical parasites (clones) were identified in *F. hepatica* from Argentina

We identified 263 distinct MLGs; with 28 of these MLGs identified more than once (Table 4). Initial analyses revealed 261 distinct MLGs and 30 multicopy MLGs, but following analyses of *P*_sex_ at the regional level, two multicopy MLGs from Corrientes were found to have *P*_sex_ > 0.05 at *n* = 2 (*P*_sex_ = 0.291 for MLG143 and MLG149; *P*_sex_ = 0.297 for MLG157 and MLG163; Supplementary Table S1). These MLGs were therefore likely to have arisen from sexual reproduction rather than asexual reproduction i.e. these were not clonemates. The remaining *P*_sex_ values were significant at *n* = 2, and ranged from 9.43 × 10^−7^ to 1.96 × 10^−12^ in Catamarca, 9.49 × 10^−7^ to 1.01 × 10^−12^ in Chubut, 0.00479 in Corrientes, and 3.49 × 10^−8^ to 7.13 × 10^−10^ in Entre Ríos. This supports the conclusion that the majority of multicopy MLGs represent parasites that arise from asexual reproduction during the clonal expansion within the snail intermediate host i.e. the majority are clonemates. Overall values for genotypic richness, a measure of the number of distinct MLGs, ranged from 0.615 to 0.984.

**Table 4:** Summary statistics following population genetic analyses of *Fasciola hepatica* across four regions of Argentina.

Parameter		Province				All locations
		Catamarca	Chubut	Corrientes	Entre Ríos	
No. of parasites successfully genotyped		27	135	62	96	320
No. of alleles	Fh_2	11	19	6	19	23
	Fh_5	10	22	4	13	25
	Fh_6	14	24	4	23	29
	Fh_10	9	10	2	10	11
	Fh_11	9	11	2	9	11
	Fh_12	5	11	6	8	12
	Fh_13	5	6	3	3	7
	Fh_15	5	9	1	7	9
No. of genotypes	Fh_2	15	49	11	52	94
	Fh_5	13	50	4	35	74
	Fh_6	13	62	5	64	116
	Fh_10	10	24	3	39	46
	Fh_11	14	27	3	25	38
	Fh_12	7	23	13	16	32
	Fh_13	8	11	3	6	12
	Fh_15	5	16	1	9	17
No. of distinct MLGs		17	98	61	87	263
No. of multicopy MLGs ^a^		6	15	1	6	28
No. of clones ^a^		16	52	2	15	85
Genotypic richness ^b, c^		0.615	0.726	0.984	0.905	ND
Gene diversity ^b, d^		0.728	0.739	0.310	0.735	ND
F_IS_ (*P* value) ^b^		0.010 (0.4734)	0.009 (0.2859)	0.041 (0.2063)	0.027 (0.0797)	ND
No. of loci pairs showing LD (*P* value < 0.05) ^b, e^		2	3	0	2	ND

^a^Only multicopy multilocus genotypes (MLGs) with *P*_sex_ values < 0.05 at *n* = 2 (i.e. clonemates) are included in these counts.

^b^Multicopy MLGs with *P*_sex_ values < 0.05 at *n* = 2 (i.e. clonemates) were reduced to one instance for these analyses (263 parasites).

^c^Genotypic richness was calculated using the formula (G – 1)/(N – 1) [where G = the number of unique MLGs and N = the number of individuals] (Dorken and Eckert, 2001).

^d^Gene diversity was averaged across loci.

^e^Linkage disequilibrium (LD) was assessed by performing pairwise tests for all 28 pairs of loci. If four or more of these were significant (*P* < 0.05) this would be a sign that there was some level of linkage disequilibrium (cumulative binomial probability = 0.049; Waples, 2015).

The majority (17) of the 28 clones were represented by two parasites (Table 5). The maximum number of clonemates representing a single clone was six (Table 5). The total number of clonal parasites was 85 (0.266; Table 4). We identified clonal parasites in 12 of the 14 definitive hosts studied, and the highest number of different clones identified in a single animal was five (Table 5). Clones/clonemates were also shared amongst hosts from the same locality (RT1, RT2 and RT3; Table 5), and from two farms (Farms 1 and 2) in distinct, but geographically close, localities (ANC and TLR; Fig. 1; Table 5).

**Table 5:** Distribution of clonemates (multicopy multilocus genotypes (MLGs) of *Fasciola hepatica* amongst the individual definitive hosts studied in Argentina.

Animal ID	No. of parasites successfully genotyped	No. of clonal parasites	No. of clonemates (parasites identified with each repeated MLG)^a^																											
			3	4	6	8	9	12	18	39	40	42	54	55	56	66	81	88	96	97	99	101	103	150	178	180	181	206	212	216
ANC	14	10	4	2	2	1	1																							
TLR	13	6	2			1	1	2																						
ARSOV	24	4							4																					
DOP	13	7								2	3	2																		
GL1	6	0																												
GL2	20	15											6	3	6															
GLOV	23	6														6														
SPCH	18	5															3	2												
SVCH	31	15																	5	4	2	2	2							
BA1	27	0																												
BA2	35	2																						2						
RT1	12	3																							1	1	1			
RT2	45	7																							1	2		2	1	1
RT3	39	5																								2	1		1	1
**TOTAL**	**320**	**85**	**6**	**2**	**2**	**2**	**2**	**2**	**4**	**2**	**3**	**2**	**6**	**3**	**6**	**6**	**3**	**2**	**5**	**4**	**2**	**2**	**2**	**2**	**2**	**5**	**2**	**2**	**2**	**2**

^a^Only multicopy MLGs with *P*_sex_ values < 0.05 at *n* = 2 (i.e. clonemates) are included in these counts and the number assigned to each MLG matches the ID codes given in Supplementary Table S1.

### 3.3 Sub-populations largely show random mating, high gene diversity and no evidence of linkage disequilibrium

We found no deviation from Hardy–Weinberg equilibrium in any province; we calculated F_IS_ and corresponding one-tailed *P* values, to assess if each sub-population deviated from Hardy–Weinberg equilibrium. All values were non-significant and ranged from 0.009 to 0.041 (Table 4). This indicates that there is largely random mating between parasites within each sub-population. Similarly, we found no evidence of linkage disequilibrium in any province; we performed unphased pairwise linkage disequilibrium tests between each pair of loci. Up to three pairs of loci were significant in each province but this is less than would be expected by chance (four or more pairs, *P* < 0.05 cumulative binomial probability = 0.049; Waples, 2015; Table 4).

The majority of regions showed gene diversity of ~0.73, however parasites from Corrientes showed a lower gene diversity of 0.310 (Table 4). This region also had a correspondingly lower number of alleles and genotypes detected, even at loci that were highly polymorphic in the other regions, e.g. Fh_6 (Table 4). Interestingly this region still showed high genotypic diversity: we identified 61 unique MLGs of the 62 parasites successfully genotyped (genotypic richness = 0.984; Table 4).

## 4 Discussion

In this study we report population genetic structuring in *F. hepatica* infecting livestock, predominantly cattle, across Argentina. None of the provinces showed evidence of deviation from Hardy–Weinberg equilibrium (F_IS_ *P* value > 0.05), so mating appears random and the amount of self-fertilisation is negligible. Movement of the definitive host is thought to represent the greatest opportunity for parasite mixing, hence reduced livestock movement due to the large geography of Argentina and the distance across which these samples were collected (up to 2250 km), may explain population structuring. Specific detail of animal movement was not known except for Corrientes, where the farm owner reported no import of livestock, effectively rendering it a closed or isolated herd, and it is interesting to note that this population showed the greatest genetic differentiation and lowest gene diversity. We cannot discount that these differences we observed at the geographical level may be due to our limited ability to detect variation amongst hosts at a local level, as we were relying on just one or two hosts per farm, as a random representation of the parasite population as a whole. In 2012, Vilas et al. reported genetic structuring of *F. hepatica* when sampling the total adult parasite burden of 10 sheep within a flock, but did not observe similar structuring in cattle populations (Vilas et al., 2012). In the study reported here, the majority of parasites originated from cattle and this may have influenced our findings of random mating. There is no standard sampling protocol to assess among infrapopulation genetic structure in a local area but Gorton et al. (2012) recommend sampling all individual parasites from 20–30 hosts when infection levels are low, or 20–30 parasites per host from 10 or more hosts when infection levels are high. This sampling approach, together with a better understanding of animal movement amongst farms, should generate greater confidence in the underlying mechanisms causing population structuring in Argentina.

Observing *F. hepatica* metacercariae directly on vegetation is not possible so it is not known if they aggregate in ‘clumps’ on pasture. However, metacercariae are known to aggregate in petri dishes (Abrous et al., 2001) and our own observations under experimental conditions suggest that they rapidly encyst on the first substrate they encounter, often congregating in large numbers (200–300 metacercariae) in small regions of visking tubing, although it is not known if this occurs on pasture. Thus, there is opportunity for extensive transmission of clones to the definitive host. We found 263 unique MLGs from the 320 parasites successfully genotyped, giving a clonal diversity of 82%. We identified genetically identical parasites (clones) in 12 of 14 definitive hosts studied, a finding consistent with previous studies in livestock, where 61% and 85% of hosts studied contained genetically identical parasites (Vilas et al., 2012, Beesley et al., 2017). This supports the theory that following clonal expansion within the snail, there is some co-transmission of clonemates to the definitive host, similar to previous reports (Beesley et al., 2017), but does not extend to the low clonal diversity observed in sheep for *F. hepatica* (Vilas et al., 2012), and it does not occur to the same extent as clonemate co-transmission recently observed for *D. dendriticum* (Criscione et al., 2020).

Generally, clonemates were found within the same host (Table 5), but we also identified clonemates shared amongst animals, for example, in Rosario del Tala, Entre Ríos clonemates were found shared amongst three animals, likely due to animals co-grazing as they originated from the same locality. However, the presence of multiple clonemates shared between two geographically close sampling sites (ANC and TLR) is novel. Since these two locations are approximately 75 km apart, it seems unlikely that these animals would have co-grazed. In the absence of further information, one explanation might be the movement/selling of livestock between the two farms (Walker et al., 2007). Another possibility would be the transfer of snails from one area to another via humans, livestock or wildlife (Mas-Coma et al., 2009; Van Leuwen, 2012. Speeding up the snail’s pace: bird-mediated dispersal of aquatic organisms. PhD thesis, Radboud University Nijmegen, Nijmegen, The Netherlands). Although we did not identify clonemates shared between different species of animals, Walker et al. (2007) recovered fluke from sheep and cattle with identical composite mitochondrial haplotypes. Most of the clones we identified in this study were represented by two parasites (clonemates), but we also identified up to six parasites with the same genotype. This agrees with previous findings of up to nine or 10 parasites with the same genotype (Vilas et al., 2012, Beesley et al., 2017), and has also been reported in *Fascioloides magna,* where nine parasites in one deer had the same five locus allozyme genotype (Mulvey et al., 1991). Within *D. dendriticum,* up to 22 parasites shared the same genotype in the second intermediate ant host (Criscione et al., 2020). However, in *S. mansoni* the mean number of copies of a clone was 3.15 (standard error ± 0.47; Prugnolle et al., 2004), and findings from aquatic trematodes usually report a maximum of two to four copies of each clone (Rauch et al., 2005, Criscione and Blouin, 2006, Keeney et al., 2007a, Lagrue et al., 2009, Leung et al., 2009, Valdivia et al., 2014).

We identified high genotypic richness and high gene diversity in the majority of regions. Three of the four provinces we studied had high gene diversity (~0.73; Table 4). However, Corrientes had a lower gene diversity of 0.310, together with lower numbers of alleles and genotypes identified at each locus, even when correcting for sample size differences (data not shown). Since there was no evidence of non-random mating, the reduced gene diversity in Corrientes could therefore be a result of a smaller effective population size. Previous analysis of parasites from three Argentinian provinces (Catamarca, Chubut and Salta) revealed only minor genetic variation (Carnevale et al., 2017). Similarly, in this study Catamarca and Chubut had the lowest pairwise F′_ST_ (0.0754) and showed lower genetic differentiation on PCoA, however these studies lacked power as they relied on small numbers of parasites (Carnevale et al., (2017) used 22 parasites, whilst in this study Catamarca was represented by 27 parasites). In Great Britain, genotypic richness of *F. hepatica* within the majority of individual definitive hosts was >0.8 (range: 0.343–1.0; Beesley et al., 2017), and hosts can acquire a genetically diverse set of parasites even over a short grazing period (Walker et al., 2011). Similarly, genotypic richness of *S. mansoni* has been reported to be 0.795 and 0.847 (Prugnolle et al., 2002, Prugnolle et al., 2004). However, amongst aquatic trematodes with two- or three-host life cycles, there is a greater ability for dispersal of clones within the environment, and the majority of genotypes within the second intermediate host or the definitive host are unique. Genotypic richness in these cases is often reported to be greater than 0.95 (Rauch et al., 2005, Criscione and Blouin, 2006, Keeney et al., 2007a, Lagrue et al., 2009, Leung et al., 2009, Valdivia et al., 2014).

For population genetic studies there is a preference for co-dominant, neutral genetic markers that allow measures of observed and expected heterozygosity such as microsatellites and allozymes (Hurtrez-Bousses et al., 2004, Vázquez-Prieto et al., 2011, Vilas et al., 2012, Cwiklinski et al., 2015, Beesley et al., 2017). Initial attempts to apply the panel of microsatellites developed by Hurtrez-Boussès et al. (2004) to *F. hepatica* in the UK were unsuccessful, necessitating development of a new panel of markers (Cwiklinski et al., 2015). It is reassuring in this regard that, although five loci (Fh_1, Fh_3, Fh_4, Fh_7 and Fh_14) had to be excluded, this work has highlighted the versatility of this microsatellite panel for analysis of the population genetic structure of *F. hepatica* from countries other than the UK. Indeed, many of the same alleles were identified in both Argentinian and British *F. hepatica* populations. Given the different patterns of population genetic structure between cattle and sheep in Spain (Vilas et al., 2012), our report here of population structuring in Argentina, and a general tendency towards clonal transmission, highlights how these microsatellites can complement and extend on existing studies to better understand *F. hepatica* transmission, and to more confidently predict development of drug resistance in *F. hepatica*.

In conclusion, the work presented here shows that there is evidence for population structuring of *F. hepatica* across Argentina and that there appears to be random mating within populations (although one must consider the caveats of our sampling approach); in addition there is evidence for some clonemate transmission.
